# Thermography imaging during static and controlled thermoregulation in complex regional pain syndrome type 1: diagnostic value and involvement of the central sympathetic system

**DOI:** 10.1186/1475-925X-5-30

**Published:** 2006-05-12

**Authors:** Sjoerd P Niehof, Frank JPM Huygen, Rick WP van der Weerd, Mirjam Westra, Freek J Zijlstra

**Affiliations:** 1 Department of Pain Treatment, Erasmus MC, University Medical Center, Dr. Molewaterplein 40, 3015 GD Rotterdam, The Netherlands; 2 Department of Anesthesiology, Erasmus MC, University Medical Center, Dr. Molewaterplein 40, 3015 GD Rotterdam, The Netherlands

## Abstract

**Background:**

Complex Regional Pain Syndrome type 1 (CRPS1) is a clinical diagnosis based on criteria describing symptoms of the disease.

The main aim of the present study was to compare the sensitivity and specificity of calculation methods used to assess thermographic images (infrared imaging) obtained during temperature provocation. The secondary objective was to obtain information about the involvement of the sympathetic system in CRPS1.

**Methods:**

We studied 12 patients in whom CRPS1 was diagnosed according to the criteria of Bruehl. High and low whole body cooling and warming induced and reduced sympathetic vasoconstrictor activity. The degree of vasoconstrictor activity in both hands was monitored using a videothermograph. The sensitivity and specificity of the calculation methods used to assess the thermographic images were calculated.

**Results:**

The temperature difference between the hands in the CRPS patients increases significantly when the sympathetic system is provoked. At both the maximum and minimum vasoconstriction no significant differences were found in fingertip temperatures between both hands.

**Conclusion:**

The majority of CRPS1 patients do not show maximal obtainable temperature differences between the involved and contralateral extremity at room temperature (static measurement). During cold and warm temperature challenges this temperature difference increases significantly. As a result a higher sensitivity and specificity could be achieved in the diagnosis of CRPS1. These findings suggest that the sympathetic efferent system is involved in CRPS1.

## Background

Complex regional pain syndrome type 1 (CRPS1) is a complication after surgery or trauma, although spontaneous development has also been described. CRPS1 is characterised by signs and symptoms of inflammation and central sensitisation. The diagnosis can be made using several different criteria sets, the most popular of which are the International Association of Pain (IASP) and the Bruehl criteria sets [1]. The IASP criteria have a high sensitivity but a lower specificity, whereas the Bruehl criteria have a high specificity but a lower sensitivity. The IASP criteria are useful for clinical aims and the Bruehl criteria appear to be more useful in research. New IASP criteria are under discussion [2] and attempts have been made to obtain a less subjective diagnosis by using diagnostic tools such as 3-phase bone scan, X-ray, MRI, fMRI, and temperature measurement devices [3]. Until now, however, none of these methods has been accepted as a gold standard. Due to the limited validity of clinical diagnoses, it may be difficult to differentiate CRPS1 from other diseases, e.g. from functional disorders with disuse. We have the impression that a false-positive diagnosis for CRPS1 is still made too often, especially in patients with complaints for which no clear explanation can be obtained regarding the onset of the symptoms.

Temperature differences are widely regarded as a predictor in the diagnosis of CRPS and videothermography has been applied as a diagnostic tool in CRPS1 [4-7]. The videothermograph is an excellent skin temperature measurement system with a high accuracy and repeatability [8]. Skin temperature is a good predictor of sympathetic activity as shown in a study in which a good correlation was found between skin temperature and skin sympathetic nerve activity [9]. Temperature at the surface of an extremity reflects the result of a complex combination of central and local regulation systems. Sherman et al. assessed the clinical usefulness of skin temperature patterns in diagnosing CRPS, by observing long-term relationships between changes in pain due to CRPS and patterns of near-surface blood flow [5]. Bruehl et al. examined the validity of thermogram derived indices of autonomic functioning in the diagnosis of CRPS; they found that temperature asymmetry accurately discriminated between CRPS and non-CRPS patients [10]. Wasner et al. evaluated the diagnostic value of skin temperature side differences as an index of induced disturbance to the sympathetic nervous system; they have shown that skin temperature differences in the distal limbs proved to be useful in distinguishing CRPS1 from other extremity pain syndromes with high sensitivity (76%) and specificity (93%) [11]. Gulevich et al. have shown a high sensitivity (93%) and specificity (89%) for stress infrared thermography in the diagnosis of CRPS; based on an estimation of 50% prior probability, the positive predictive value was 90% and the negative predictive value was 94% [6]. However, the findings of these studies are not consistent with respect to sensitivity, specificity and reliability, probably because different analysing schemes were used to assess the thermographic data. Other factors possibly influencing the discriminating power of temperature measurement in CRPS1 is the cyterian cycle of the sympathetic system. Wasner and colleagues showed an improvement in sensitivity and specificity using temperature measurement of fingertips during cold and hot challenge [12,13]. Nowadays, the infrared tympano thermometer is very popular to measure skin temperature in CRPS1. The temperature should be measured in a matrix of representative points, as described by Oerlemans et al. [14]. The average difference between the involved and contralateral extremity is calculated using the points defined by the matrix. In an earlier study we demonstrated improved sensitivity and specificity of temperature measurement when using computerized videothermography at room temperature and also introduced the asymmetry factor [15].

The main aim of the present study was to compare the sensitivity and specificity of calculation methods used to assess thermographic images (infrared imaging) obtained during cold and warm temperature provocation. The secondary objective was to obtain information about the involvement of the central sympathetic system in CRPS1.

## Methods

### Patients and controls

This study was approved by the local Medical Ethical committee of the Erasmus Medical Centre.

From April (spring) 2003 through September (autumn) 2003 we included 12 patients, (11 women and 1 man) with a mean age of 51.5 (range 37–66) years. All patients gave written informed consent. A physician with considerable experience in diagnosing and treating CRPS1 (FJPM), included the patients according to the Bruehl criteria [1]. Only patients with a unilateral CRPS1 in the upper extremity were included. During the same period we studied 8 healthy volunteers (control) without a history of neurotrauma and/or vascular disease (5 women and 3 men) with a mean age of 29.4 years (range 22–48) years. Data on the patients are presented in Table 1.

**Table 1:** Clinical characteristics of the CRPS1 patients

Subject no.	Age(years)/sex	Time since Onset of disease (months)	Dominant side	Location of CRPS1	Precipitating event
1	43/f	10	Left	Left	removal of tumor digit 3
2	49/f	8	Right	Right	Colles fracture
3	52/f	8	Right	Left	unknown trauma
4	51/f	6	Left	Right	tendon trauma
5	63/f	5	Right	Right	Colles sprain
6	56/f	8	Right	Right	Colles fracture
7	66/f	9	Right	Right	Colles fracture
8	41/f	10	Right	Left	Colles fracture
9	47/f	3	Right	Left	injection into wrist
10	56/f	3	Right	Right	arthrose digit 1
11	57/f	3	Right	Left	Colles fracture
12	37/m	6	Left	Left	Colles fracture
Average	51.5	6.6			
SD	8.7	2.6			

### Power calculation

Data from our previous study were used to perform a power calculation; in that study the difference between the asymmetry factor of patients and controls was 0.41 and the combined SD was 0.31 [15]. Considering a ratio between patients and controls of 0.65 with a significance of 0.05, for the present study the number of patients needed to obtain 80% power was 12 and the number of controls needed was 8.

### General questionnaires and measurements

The severity of the CRPS1 is estimated using the total Impairment level Sum Score (ISS). The ISS total is the sum of the Visual Analogue Scale (VAS), The McGill pain questionnaire (MPQ), active range of motion (AROM), edema and temperature difference that are converted to ISS scores (ranging from 1–10). The VAS is a reliable and valid instrument to measurement pain intensity [16], with scores range from 0 (no pain) to 10 (most intense pain). The VAS is used to measure the momentary pain during rest, and pain during the cold and warm temperature cycle.

The MPQ, Dutch language version, is a reliable and valid tool to measure the amount of pain in a variety of complaints [17];

The AROM was used to reflect physical dysfunction. Scores from both the unaffected hand and the affected hand were measured, and the differences in range of motion from five joints were recorded (range 1–5 points per joint, 5 points for maximal limitation). The presence of edema in the affected limb was measured in comparison with the unaffected hand. The percentage differences in volume were determined after successive immersion of both hands in a tube containing water at approximately 30°C. The amount of displaced water was weighed on-line using a laboratory balance (Sartorius, Breukelen, the Netherlands; accuracy 1 g), based on the method described by Fereidoni et al. [18].

To comply with the standard ISS score defined by Oerlemans et al. [19]., the skin temperature of both hands was measured by a tympanic thermometer (M3000A, First Temp Genius^®^, Tyco Healthcare Ltd, Gosport, UK). The thermal sensitivity of the thermometer is 0.05°C at 30°C. Five measuring points on both extremities were marked using a predefined matrix.

All the outcomes above were then converted to a score ranging from 1 to 10, resulting in an ISS total with a minimum score of 5 and a maximum score of 50. A score of 5 indicates the least level of impairment and 50 the highest level of impairment. The ISS total is the sum of the VAS, the MPQ, AROM, edema and temperature difference that are converted to ISS scores (ranging from 1–10), as described by previously by Oerlemans et al. [19].

### Temperature challenge to experimental condition

The most effective way to alternate sympathetic vasoconstrictor activity is by whole body warming and cooling [20]. To achieve whole body warming and cooling a thermosuit was used (Thermowrap, MTRE, Akiva Industrial Park, Israel). During the experiment the room temperature was kept constant at 23 ± 0.5°C. The thermosuit did not cover the head, hands and feet of the subjects. The thermosuit, connected to a thermostatic pump (Ecoline, Lauda, Lauda-Königshofen, Germany), is pivoted by water channels and in direct contact with the skin providing a high-energy transfer. The water inflow temperature was set to a temperature of 15 ± 1.5°C during the cold cycle, and 45 ± 1°C during the warm cycle. The flow of water was 2 l/min, effectively replacing the water in the suit almost every minute. This method allows to achieve a controlled sympathetic activation of the vasoconstrictor neurons supplying the hands and feet [13]. Maximum vasoconstrictor activity was presumed to be reached when the mean temperature of the fingers of the contralateral side achieved room temperature during the cold cycle. Minimum vasoconstrictor activity was presumed to be reached when the average fingertip temperatures of the contralateral side was at 96% of the tympanic temperature measured at baseline. In the control group the fingertip temperature of the dominant side was considered as an indicator of maximum and minimum vasoconstrictor activity.

### Temperature measurement

Skin temperature of both hands was measured with a computer-assisted infrared thermograph (ThermaCam SC2000, FLIR, Danderyd, Sweden). This thermographic camera produces a matrix (representing image points) of temperature values. The thermal sensitivity of the thermograph is 0.05°C at 30°C. The spectral range is 7.5–13 μm and the built-in digital video is 320×240 pixels (total 76,800 pixels). Data were obtained through a high-speed (50 Hz) analysis and recording system coupled with a desktop PC (ThermaCAM Researcher 2001 HS). Because calculation on the thermograms took place after the experiment, the thermograms were stored on a hard disk (14-bit resolution) awaiting analysis. The emissivity of the skin was set in the software to 0.98 and the apparent temperature was measured and also set in the software. Before each recording the camera was calibrated using the system's internal calibration of the software connected to the camera.

### Baseline measurements

Baseline differences between the two hands were measured at room temperature (23°C), with patients and controls kept in an upright position for 15 min to obtain sympathetic equilibrium with the surrounding (resting conditions). A thermographic image of the dorsal side of both hands was taken parallel to the hand from a distance of 70 cm incorporating the whole hand including the wrist, based on the method described Huygen et al. [15]. Thereafter, the VAS scale was used to record the pain at that moment, the MPQ was filled in, and the AROM and hand volume were measured.

### Measurements during the cold cycle

**Figure 1:**
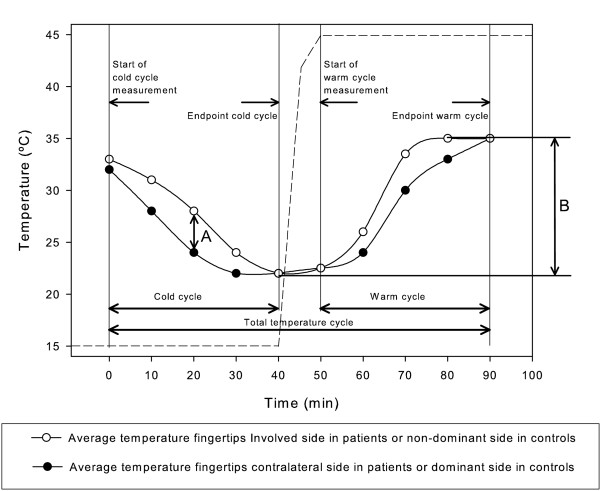
**A hypothetical temperature cycle and terms used in this study**. A; maximum difference between fingertip temperature, B; temperature span during total temperature cycle.

After the baseline measurements the subjects put on a bathing suit/trunk, then lay in a supine position in the thermosuit (connected to a thermobath), which was already cooled to 15°C, resulting in a massive sympathetic vasoconstriction activity. Immediately temperature measurements took place every 10 min until all fingertips on the contralateral side reached room temperature (23°C) (see Figure 1, Cold cycle). In the control group the dominant side was considered as reference. The temperature measurements were made at 70 cm distance parallel to the hand, using a tripod to hold the videothermographic camera. The tympanic temperature of patients and controls was measured at the end of the cold cycle. Patients were asked to indicate the pain level every 10 minutes during the entire cold cycle by means of a VAS score.

### Measurements during the warm cycle

When the cold cycle was completed, the thermo bath was emptied and refilled with water heated to 40°C. This resulted in a suit temperature of 40°C within approx. 5 min while the subjects were kept in the suit. Then the temperature was set to 45°C, which was reached within 5 min. The temperature of 45°C leads to a low sympathetic vasoconstrictor activity. From then onwards temperature measurements took place every 10 min until all fingertips on the uninvolved side reached 96% of the tympanic temperature (Figure 1, Warm cycle). Recording of the thermographic images took place in the same way as during the cold cycle. Patients were asked to indicate their pain level during the entire warm cycle by means of a VAS score every 10 min.

## Calculations

### Calculation

The temperature information in the thermographic images taken at baseline, and during the cold cycle and warm cycle, contain both the environmental temperature and the temperature of the hand. A threshold temperature was used to filter out the environmental temperature. Calculation of the fingertip temperature and the asymmetry factor was used to describe the sympathetic vasoconstrictor state every 10 min.

### Average fingertip temperature

Using software, spots of approximately 30 pixels were placed on the fingertips representing 19 mm^2 ^of the fingertips (Thermacam researcher software 2000). The average temperature of a spot was considered to be a representative temperature of a fingertip. Then, for each hand, the average fingertip temperature was calculated by averaging over all 5 spots. In patients, the absolute difference in fingertip temperature was calculated by subtracting the average fingertip temperature of the involved hand from the average fingertip temperature of the contralateral hand. The same absolute difference fingertip temperature was calculated in controls where the fingertip temperature of the dominant hand was subtracted from the non-dominant hand.

### Asymmetry factor

This method determines the asymmetry factor (correlation) between the temperature histogram of the involved and contralateral extremity based on the method described by Huygen et al. [15]. The asymmetry factor is a factor describing the degree of dissimilarity between temperature data obtained from one hand compared to the other hand. A score of 1 indicates the same temperature distribution; a lower score indicates less similarity. This calculation was applied to the thermographic recording of both patients and controls.

### Discriminating power and vasomotor activity

To assess whether the temperature provocation increased the discrimination between patients and controls the following selection was used:

For the fingertip temperature; the absolute average fingertip temperature difference at baseline was compared to the maximum absolute difference between the average fingertip temperature that was reached during the total temperature cycle (Figure 1A).

For the asymmetry factor; the asymmetry factor at baseline was compared to the minimum asymmetry factor that was reached during the temperature cycle.

To compare vasomotor activity span between the involved and contralateral hand, the differences between the minimal fingertip temperature and the maximal fingertip temperature during the whole temperature cycle were calculated in both the involved and contralateral hand (Figure 1B)

The maximum VAS pain rating that was present during the total temperature cycle was also selected in each subject.

### Calculating the sensitivity, specificity, positive predictive value and negative predictive value

The receiver operating characteristic (ROC) curve is a very good indicator of the discriminating power of a diagnostic method. The coordinates of the plot are defined by calculating the sensitivity and specificity at different values of the diagnostic test, called cut-off points. The sensitivity is plotted on the vertical axes and specificity is plotted as 1 minus the specificity on the horizontal axes. This results in a plot of the true-positive rate against the false-positive rate for the different possible cut-off points in a diagnostic test. The area under the ROC curve (AUC) is a measure of the accuracy of the diagnostic test used. The accuracy is measured on a five-point scale: excellent (area of 1-0.9), good (area of 0.9-0.8), fair (area 0.8-0.7) poor (area of 0.7-0.6), and fail (area of 0.6-0.5) [21,22]. In assessing a diagnostic test the positive and negative predictive value is needed. The positive predictive value is the proportion of patients with positive test results who are correctly diagnosed; the negative predictive value is the proportion of patients with negative test results who are correctly diagnosed. The positive likelihood ratio is the ratio between true-positive and true- negative; the negative likelihood ratio, is the ratio between false-positive and true- negative. A summary of the above is given in Table 2.

**Table 2:** Calculation of sensitivity, specificity, positive predictive value and negative predictive value.

		CRPS1 according to the Bruehl criteria.			
		
		Positive	Negative	Total	Calculations
CRPS according to thermographic results	Positive	A	B	A+B	
	Negative	C	D	C+D	
	Total	A+C	B+D	N	
	Calculations				

### Statistical analysis

For comparison of the non-parametric data between CRPS1 patients and healthy controls, the Mann-Whitney *U *and Spearman tests were used. Data are given as median and interquartile range (IRQ). A *p-*value of <0.05 was considered statistically significant. These analyses were performed with the SPSS^® ^10.1 software package.

To compare the discriminating power of baseline temperature measurements with measurements of temperature during the temperature cycle, a ROC analysis was performed. The ROC curve was calculated at baseline, and using the values obtained during the temperature cycle as described earlier. Statistical comparison of the ROC curves was performed using the software program ROCkit 0.9, which incorporates a method developed by Metz et al. to compare correlated ROC curves [23]. In the present study specificity is used to indicated the discrimination between CRPS patients and healthy controls.

## Results

To compare the average fingertip temperature and the asymmetry factor with respect to their diagnostic value, the following results were used in performing the ROC analysis.

### Median average fingertip temperature

**Figure 2:**
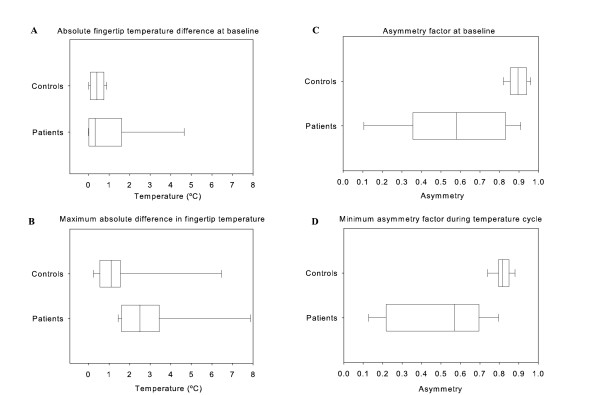
**Differences between controls and patients at baseline and during the temperature cycle**. A, the absolute average temperature difference between fingertips at baseline. B, the maximum absolute average temperature difference between fingertips during the cycle. C, the asymmetry factor at baseline. D, the minimum asymmetry factor during the temperature cycle.

At baseline, the difference in absolute fingertip temperature difference in CRPS1 patients and controls between involved and contralateral extremities, or in controls between the dominant and non-dominant extremity, was calculated (Figure 1 zero min). At baseline, the median fingertip temperature difference for controls was 0.43°C (0.04–0.66°C) and for patients was 0.37°C (0.10–0.77°C) (Figure 2A).

During the total temperature cycle, the maximum difference in absolute fingertip temperature difference in patients and controls between involved and contralateral extremities, or in controls between the dominant and non-dominant extremity, was calculated (Figure 1A). During the total temperature cycle the average fingertip temperature in controls was median 0.95°C (0.50–1.51°C) and in patients median 2.50°C (1.61–3.43°C) (Figure 2B).

### Asymmetry factor

The asymmetry factors at baseline, and the minimum asymmetry factor obtained during the vasoconstrictor changes, were calculated. At baseline, for the controls the calculated asymmetry median was 0.89 (0.85–0.94) and for CRPS patients was 0.58 (0.36–0.83) (Figure 2C).

During the temperature cycle, for controls the calculated minimum asymmetry median was 0.82 (0.79–0.87) and for patients was 0.56 (0.22–0.70) (Figure 2D).

### ROC analysis

The results above were used in the ROC analysis. The small difference in fingertip temperature difference at baseline between patients and controls resulted in a low sensitivity and specificity, with a low AUC. The sensitivity and specificity improved when using the data obtained from the temperature cycle. In comparing the ROC curve of the baseline measurement with the ROC curve obtained from the temperature cycle a significant improvement in favor of the temperature cycle was calculated.

The clear difference in asymmetry factors at baseline and during vasoconstrictor activity results in a higher sensitivity and specificity with a higher power (Figure 2 A1, B1, C1 and D1). Comparison the ROC curve at baseline with the ROC curve obtained during the temperature cycle showed no significant improvement in sensitivity and specificity (Table 3).

**Table 3:** Sensitivity and specificity at baseline and during cold/warm cycle of temperature difference and the asymmetry factor

	|Average fingertip temperature difference at rest.|	|Max. average fingertip temperature difference during temperature cycle.|	Asymmetry factor at rest.	Minimum Asymmetry factor during the temperature cycle.
Sensitivity	76%	92%	100%	100%
Specificity	38%	75%	75%	83%
Positive predictive value	62	85	100	100
Negative predictive value	43	86	73	79
Cut-off points	0.1°C	1.4°C	0.81	0.73
AUC 95% interval and Std.Error	0.48 0.221–0.737(0.101)	0.87* 0.666–1.063(0.101)	0.90* 0.767–1,035(0.068)	0.96** 0.879–1.038(0.40)
Likelihood ratio positive test	1.2	3.7	4	5.9
Likelihood negative test	0.6	0.1	0	0
Difference in AUC	0.39*		0.06	

*Significant at the p < 0.05 level.

**Significant at the p < 0.001 level.

### Vasomotor activity in CRPS1 and controls

**Figure 3:**
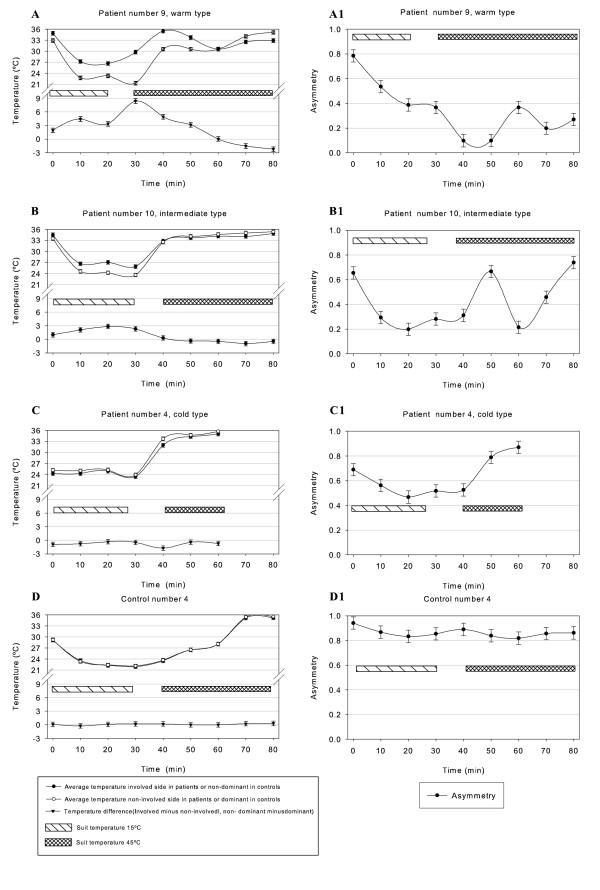
**Representative graphs of temperature and asymmetry of three patients and one control**. Left column A, B, C, D presents graphs of average temperatures of fingertips and temperature difference between the finger tips during the warm and cold temperature cycle in the three regulation types. Right column A1, B1, C1, D1 asymmetry factor during the warm and cold temperature cycle in the three regulation types.

**Figure 4:**
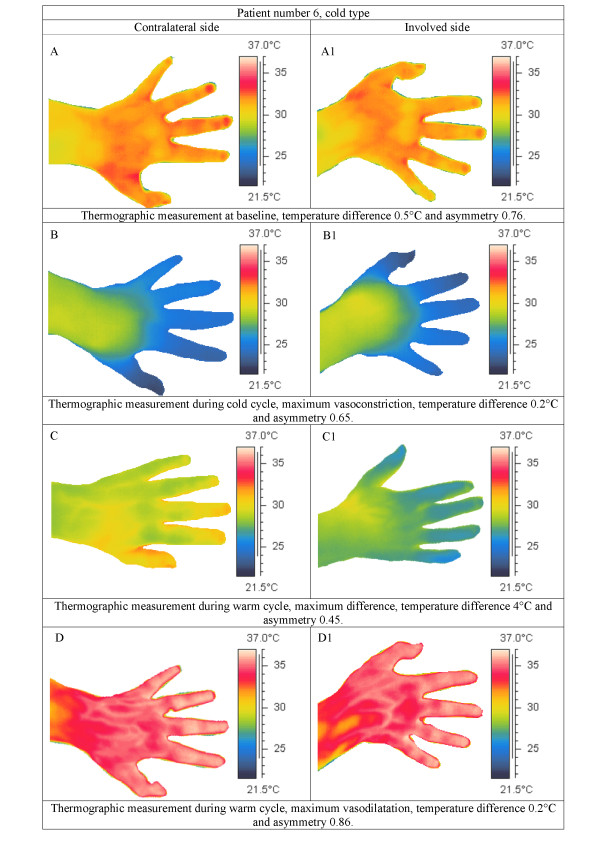
**Representative thermographic images of a patient during the temperature cycle**. Left column, images of the contralateral side. Right column, images of the involved side. A, A1 baseline recordings. B, B1 maximum vasoconstriction. C, C1 maximum temperature difference. D, D1 maximum vasodilatation.

Figure 3 presents representative examples of average temperature measurements in three patients and in one control subject. Figure 4 presents the thermographic images of one representative patient during the temperature cycle.

### Average fingertip temperature

Whole body cooling induced a maximal vasoconstrictor activity resulting in a lower flow rate, which in turn resulted in a lower fingertip temperature during cooling activity. After this, whole body warming was performed to completely inhibit the cutaneous activity; this resulted in a higher blood flow rate, which led to an increase in the fingertip temperature. The difference in hand temperature in controls showed a minimal difference over the whole temperature cycle (for an example of a control subject see Figure 3D), whereas the difference in average fingertip temperature in CRPS1 patients is not as constant as in controls (Figure 2 and Figure 3 A, B and 3C).

To ensure the minimum vasoconstrictor activity was reached in both patient and control subjects, the average fingertip temperature of the contralateral hand in patients and the dominant hand in controls were compared at the end of the cold cycle (Table 4). To ensure that maximum vasodilatation was reached in both patient and control subjects, the average fingertip temperature of the contralateral hand in patients and the dominant hand in controls were compared at the end of the warm cycle (Table 4).

**Table 4:** Minimum and maximum fingertip temperature obtained in patients and controls at the beginning and end of the temperature cycle.

	Minimum median average fingertip temperature		Maximum median average fingertip temperature	
	
	Contralateral (patients) or dominant (controls)	Involved (patients) or Non- dominant (controls)	Contralateral (patients) or dominant (controls)	Involved (patients) or non -dominant (controls)
Patients	23.5 (22.5–24.0)	23.9(22.8–25.6)	35.0(34.3–35.7)	35.1(34.6–35.7)
Controls	23.2(22.2–24.4)	23.1(22.8–23.5)	35.1(34.4–35.2)	35.1(34.1–35.4)

### Cold, intermediate and warm regulation type classification scheme

Patients are classified into three types of regulation. The warm regulation type in whom the involved side more often had a higher in temperature than the contralateral side (Figure 1A). The intermediate type, in whom the temperature difference between the fingertips of the involved side was as often high as was low during the total temperature cycle (Figure 2B). The cold regulation type in whom the fingertip temperature of the involved side was more often lower than higher during the whole temperature cycle (Figure 2C). This resulted in 5 regulation types classified as warm, 5 types classified as intermediate and 2 regulation types classified as cold.

### Differences in temperature span and asymmetry factor during cold and warm cycle

Data from the 5 warm regulation types and the 2 cold regulation types gave an indication concerning differences between these regulation types. In the regulation types that were classified as warm, the difference between the lowest and highest temperature of the involved side was higher in comparison to the contralateral side. In regulation types that were classified as cold, the difference between the lowest and highest temperature of the involved hand was lower in comparison to the contralateral side. Because only 5 warm and 2 cold regulation types could be identified, no statistical tests were performed on these data.

From the 5 warm regulation types, 4 patients showed the largest temperature differences during the warm cycle and 1 during the cold cycle. Of the 2 cold regulation types 2 showed the largest temperature difference during the cold cycle.

### Classification of patients

The severity of CRPS1 in patients was assessed using parameters describing pain, immobility, temperature, MPQ and volume; the results are presented in Table 5.

**Table 5:** Disease activity scores of CRPS1 patients.

Age(Years)	Disease duration (months)	VAS (0–10)	MPQ (0–10)	AROM (0–10)	Vol. Diff (0–10)	Temp.diff (0–10)	Total ISS (%)
51.5(44.0–56.8)	6.0(3.0–7.5)	5.0(3.0–6.0)	5.5(2.0–8.0)	5.5(3.0–7.0)	2.5(2.0–4.0)	3.0(1.0–4.0)	46(30.0–50.0)

Based on Oerlemans et al. [19] and percentage of total score for patients with complex regional pain syndrome type 1 (CRPS1).

VAS visual analogue pain scale, MPQ McQill Pain Questionnaire, AROM active range of motion, Vol.Diff. volume difference between the contralateral hand and the involved hand, Temp.Diff. temperature difference between average temperature measured with tympanometer in the contralateral and involved hand, ISS impairment level sum score.

Results are presented as median (IRQ)

In the present series of 12 patients the correlation between the ISS total and asymmetry at rest was R = -0.678 p = 0.015, the correlation between disease duration and asymmetry at baseline was R = -0.634 p = 0.027, and the correlation between the maximum VAS rating and minimum asymmetry factor was R = -0.622, p = 0.019.

## Discussion

In this study a thermographic camera was used to assess the results of changes in temperature in CRPS. High and low sympathetic vasoconstrictor activity was induced by whole body cooling and warming in 12 patients and in 8 healthy controls. The degree of vasoconstrictor activity in the hands was monitored by skin temperature measurement using videothermography. The acquired images were subsequently used to calculate the average temperature difference and the asymmetry factors at baseline (static measurement), and during exposure to 15°C and 45°C surrounding temperature (dynamic measurement), respectively. The relation between thermography and the factors describing the disease activity was calculated. The ROC was used to assess the discriminating power of thermography in combination with different calculation methods.

During vasoconstrictor alternations we found an increase of 2.13°C in the median temperature difference between the involved and contralateral side; furthermore, the median asymmetry decreased from 0.57 to 0.56. As a result, the discriminating power of the average temperature difference increased significantly, whereas there was no significant increase in the discriminating power of the asymmetry calculation. The largest studied population, performed by Veldman in 1993 showed that in 829 patients only 39 patients had CRPS in more than one limb, 34 patients in two limbs, 4 in three limbs and 1 patient in all four limbs [27]. During cold and warm stress cycles no significant differences in fingertip temperatures between the involved and contralateral hands were found at maximal cooling (approx. 24°C, Table 4) or at warming up (approx. 34°C, Table 4), and no differences were found between controls and patients.

Our previous study [15] showed that average calculations on thermographic data are not the most accurate calculation method for diagnostic purposes in CRPS1 patients; therefore we postulated a new mathematic approach. This resulted in a sensitivity of 92% and a specificity of 94% [15]. Wasner et al. reported that the maximum temperature difference during external temperature provocation resulted in mean temperature differences of 4.5 ± 0.6°C in CRPS1 patients and of 1.3 ± 0.1°C in controls [12]. Although in this study no values of baseline measurements were reported [12], in another study by Wasner et al. these values where reported as median 1.8°C and range 0–9.4°C [11]. In the present study the maximum temperature difference during external temperature provocation in CRPS1 patients resulted in a maximum median temperature difference of 2.5°C (1.61–3.43°C), whereas at baseline this difference was 0.37°C (0.10–0.77°C). For controls this temperature difference increased from 0.43°C (0.04–0.66°C) to a maximum of 0.95°C (0.50–1.51°C). Therefore, the present results reproduce and confirm the results of the previous studies by Wasner and colleagues [7,10-12]. Although we did not include other types of diseases in this study Wasner et al. have previously demonstrated that the obtained temperature difference in CRPS patients is very specific for this patient group [12].

Temperature measurements have been studied in relation to diagnosing and monitoring CRPS. Sympathetic vasoconstrictor patterns were found to be affected in CRPS [7,24-26]; these studies indicate that the central sympathetic system does affect vasoconstrictor activity and is involved in CRPS. Although in the present study we have included the minimum number of subjects as calculated by the power calculation, the small number of patients could be a limitation of this study. The use of a contralateral extremity as a control can, theoretically, produce some problems. CRPS can have a small spread. Epidemiological studies on CRPS show a huge range in incidence. The largest studied population, performed by Veldman showed that in 829 patients only 39 patients had CRPS in more than one limb, 34 patients in two limbs, 4 in three limbs and 1 patient in all four limbs [27]. In the case we would have included a patient with a CRPS in the contralateral side this would have had a negative influence on the outcome. This confirms that at least two pathways could be involved in CRPS1: 1) peripheral inflammation, which generally increases temperature, and 2) disturbances in central temperature regulation, which could result in a changed (local) temperature of the injured extremity.

Previous studies mainly used baseline ('static') measurement and did not provoke vasoconstrictor activity. One study, with a set-up similar to ours, investigated the dynamics of the sympathetic system in CRPS1 patients [11-13,28,29]. These authors provoked the sympathetic system and found that during resting conditions a CRPS patient does not show the maximum temperature difference between extremities. The studies mentioned above used a spot thermometer or a spot blood flow meter, which are only able to measure a small area of the extremity thereby neglecting increased temperature at other locations. Furthermore the obtained data were only subjected to calculations on the average temperature, thereby possibly minimizing temperature peak values.

## Conclusion

In the present study the sensitivity had a value of 100% and the specificity a value of 75%. The results during temperature provocation revealed a sensitivity of 100% and a specificity of 83% with an increased AUC, indicating considerable improvement as a diagnostic tool. Furthermore, because of the difference between sensitivity and specificity obtained from average fingertip temperature in favor of the sensitivity and specificity obtained using the asymmetry factor, the conclusion must be drawn that temperature measurement of the fingertip alone is not sufficient.

In the present study we found no evidence that the warm type CRPS1 patients would show lower asymmetry factors during the cold cycle, or that the cold type CRPS1 patients would show higher asymmetry factors. Because the patient population of the present study consisted of 5 warm type, 5 intermediate type and 2 cold type CRPS1 patients, no conclusion about the above-mentioned effect could be drawn. However, some remarkable differences in patterns of temperature regulation were observed between the warm and cold type patients (Figure 4). Therefore, the cold and warm stress test is useful to differentiate between "warm" and "cold" type CRPS1.

The difference in temperature span in cold and warm type CRPS patients is another strong indicator that this disease is partially caused by central deregulation. This conclusion promotes the use of thermography as an objective monitoring tool in an intervention study, in order to reveal the contribution of both the central and peripheral regulation systems.

In summary, there was a significant increase in the difference in fingertip temperature between patients and controls during vasoconstrictor alternations in CRPS1 patients. However, this increase in discriminating power was not present when using the asymmetry factor. This indicates that baseline temperature measurement of the fingertips alone is not sufficient for diagnostic purpose. Instead, the temperature should be measured at various locations on the hand.

## Competing interests

The author(s) declare that they have no competing interests.

## Authors' contributions

SPN participated in the design of the study, performed the experiment, performed the statistical analysis and drafted the manuscript. FJPMH participated in the design, coordination and inclusion of patients and helped to draft the manuscript. RWPW helped with inclusion of patients and assisted with measurement of patient during the experiment. MW helped with the experiment and performed the calculation on the thermographic images. FJZ participated in the design and coordination of the study and helped to draft the manuscript.
